# Gene expression analysis reveals mir-29 as a linker regulatory molecule among rheumatoid arthritis, inflammatory bowel disease, and dementia: Insights from systems biology approach

**DOI:** 10.1371/journal.pone.0316584

**Published:** 2025-01-15

**Authors:** Devi Soorya Narayana S., Vino Sundararajan

**Affiliations:** Integrative Multiomics Lab, School of Bio Sciences and Technology, Vellore Institute of Technology, Vellore, Tamil Nadu, India; University of Vermont College of Medicine, UNITED STATES OF AMERICA

## Abstract

**Background:**

Rheumatoid arthritis (RA) is a degenerative autoimmune disease, often managed through symptomatic treatment. The co-occurrence of the reported extra-articular comorbidities such as inflammatory bowel disease (IBD), and dementia may complicate the pathology of the disease as well as the treatment strategies. Therefore, in our study, we aim to elucidate the key genes, and regulatory elements implicated in the progression and association of these diseases, thereby highlighting the linked potential therapeutic targets.

**Methodology:**

Ten microarray datasets each for RA, and IBD, and nine datasets for dementia were obtained from Gene Expression Omnibus. We identified common differentially expressed genes (DEGs) and constructed a gene-gene interaction network. Subsequently, topology analysis for hub gene identification, cluster and functional enrichment, and regulatory network analysis were performed. The hub genes were then validated using independent microarray datasets from Gene Expression Omnibus.

**Results:**

A total of 198 common DEGs were identified from which CD44, FN1, IGF1, COL1A2, and POSTN were identified as the hub genes in our study. These hub genes were mostly enriched in significant processes and pathways like tissue development, collagen binding, cell adhesion, regulation of ERK1/2 cascade, PI3K-AKT signaling, and cell surface receptor signaling. Key transcription factors TWIST2, CEBPA, EP300, HDAC1, HDAC2, NFKB1, RELA, TWIST1, and YY1 along with the miRNA hsa-miR-29 were found to regulate the expression of the hub genes significantly. Among these regulatory molecules, miR-29 emerged as a significant linker molecule, bridging the molecular mechanisms of RA, IBD, and dementia. Validation of our hub genes demonstrated a similar expression trend in the independent datasets used for our study.

**Conclusion:**

Our study underscores the significant role of miR-29 in modulating the expression of hub genes and the associated transcription factors, which are crucial in the comorbidity status of RA, dementia, and IBD. This regulatory mechanism highlights miR-29 as a key player in the pathogenesis of these comorbid diseases.

## 1. Introduction

Rheumatoid Arthritis (RA) is a persistent systemic autoimmune disorder distinguished by a myriad of clinical manifestations and an unpredictable course, with a global prevalence of approximately 0.5–1% [1]. The disease primarily involves synovial inflammation, majorly affecting the joints bilaterally and leading to symptoms of pain, swelling, stiffness, and progressive fatigue [2]. Despite the extensive ongoing research, the precise etiology of RA remains elusive. However, evidence suggests a complex interplay of genetic predispositions, supported by familial clustering and environmental triggers such as smoking and certain microbial infections may initiate or exacerbate the autoimmune response [3, 4]. Despite the chronic nature of the condition and its potential for associated comorbidities, advances in treatment strategies have significantly enhanced survival rates and the quality of life for RA patients, underscoring the importance of timely diagnosis and targeted treatment to achieve optimal disease control and reduce long-term complications [5].

Recent findings indicate that RA extends well beyond joint inflammation, impacting various organ systems and elevating the risk of several comorbidities, including cardiovascular complications, specific cancers (lymphoma, colon, and lung), depression, infections, and respiratory complications [6]. Increasingly, research emphasizes the systemic nature of RA, identifying the extra-articular comorbidities such as dementia, and IBD as significant to patient prognosis [6]. Dementia, a leading neurological complication, has recently been linked to RA, with studies showing a 1.61-fold increased risk among RA patients [7]. This association may stem from chronic systemic inflammation affecting the brain, leading to microglial activation and cytokine-driven neuronal damage, as supported by both in vitro and population-based epidemiologic studies [8]. Furthermore, the inflammation can disrupt the vascular supply to key organs including the brain, potentially heightening dementia risk. It is also reported that some medications used in RA management may also contribute to this susceptibility, although tumor necrosis factor (TNF) inhibitors show promising results in decreasing dementia risk [9]. Interestingly, Min C et al. (2020) observed no substantial associations between RA and dementia in a Korean cohort, highlighting the need for further research across diverse populations [10]. In contrast to dementia, the comorbid relationship of IBD with RA is well documented [11–13]. Both autoimmune diseases, share common immunological pathways, overlapping genetic markers, and antigens contributing to their clinical interplay [13]. Histopathological analyses also reveal that, despite affecting distinct organs and tissues, the diseases have similarities such as chronic inflammation and immune cell infiltration, cytokine-mediated pathology, tissue remodeling and fibrosis, and autoantibody production [14].

**Fig 1:**
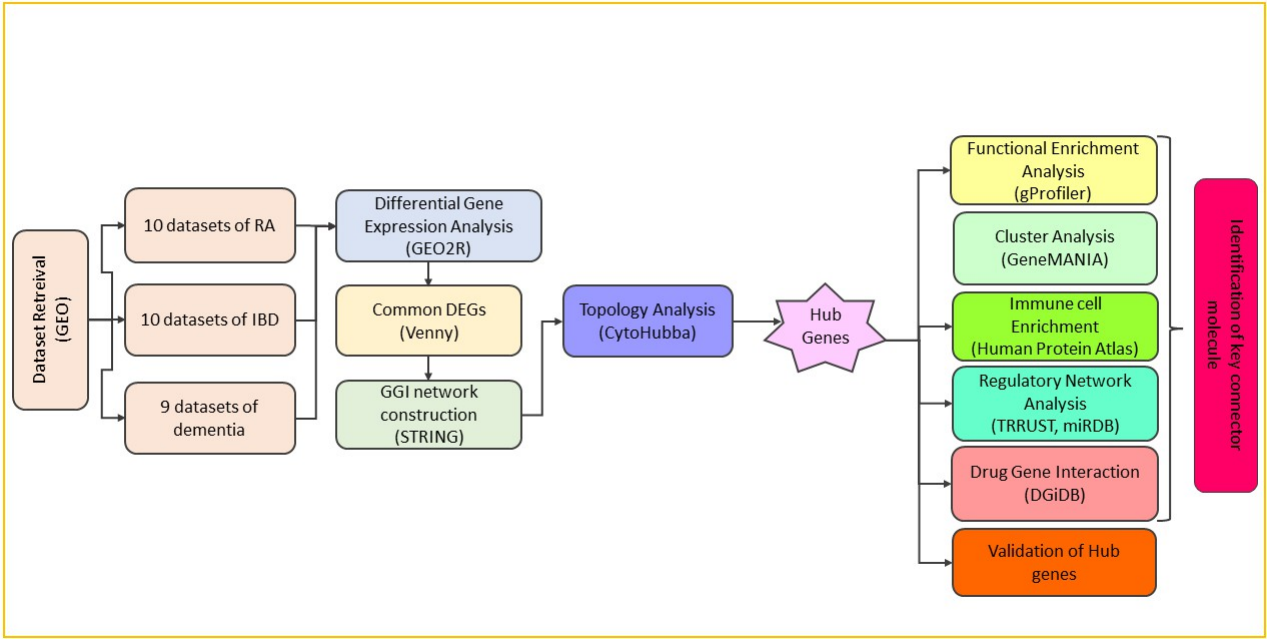
The schematic diagram representing the workflow of the study.

By utilizing systems biology-based gene expression analysis, this research aims to uncover shared biomarkers or a molecular link between RA, IBD, and dementia, which might help in pinpointing potential therapeutic targets and explore the opportunities for drug repurposing that could address the overlapping pathological mechanisms in these diseases (Fig 1). The significance of this study lies in the potential for advancing the management of the three conditions marked by chronic inflammation and immune dysregulation. Also, this study might potentially accelerate the development of effective therapies that simultaneously address the inflammatory processes in RA, IBD, and dementia, and reduce the burden of these comorbid conditions, which could ultimately improve the outcomes and quality of life for the patients.

## 2. Materials and methods

### 2.1 Data collection

We extracted microarray gene expression datasets pertinent to RA, IBD, and Dementia from the Gene Expression Omnibus database (GEO) (https://www.ncbi.nlm.nih.gov/geo/). Utilizing the search keywords “Rheumatoid Arthritis,” “Inflammatory Bowel Disease,” and “Dementia,” consecutively, the retrieved datasets were filtered employing the term “homo sapiens” to ensure relevance to human biology [15]. Our study established rigorous inclusion criteria, necessitating that (i) datasets incorporate samples from both disease and control groups, (ii) expression profiling employs array sequencing technique, (iii) samples originate from human cohorts, (iv) considering only Alzheimer’s disease (AD) samples under the umbrella of dementias considering their incidence and prevalence and (v) each dataset encompasses a minimum of 10 samples [16, 17]. The comprehensive details of the datasets incorporated in this investigation are presented in S1 Table in S1 File, facilitating transparency and reproducibility of our analyses. Three datasets GSE68689, GSE9452, and GSE140829 were selected as validation datasets for RA, IBD, and dementia respectively.

### 2.2 Identification of Differentially Expressed Genes (DEGs)

The DEG analysis was performed to discern genes exhibiting significant differences in expression levels between sample and control data within each dataset. One major concern in our study was the selection of a relevant method for DEG analysis. Though methods like Rank product and EBSEq have particular strengths in DEG identification, they are not implemented in this study considering the platform variability and large methodological adjustments required in our datasets. The GEO2R online tool, aligned with the principles of the limma Bioconductor package, was employed to identify DEGs after normalizing the raw gene expression from the expression datasets [18]. To enhance the robustness of our analyses, outliers within the datasets were identified and subsequently removed through log2 transformation and consideration of p values [19]. The DEG analysis, pivotal to our investigation, was conducted by integrating the false discovery rate-adjusted p-value using Benjamini- Hochberg correction, yielding a comprehensive table encompassing gene symbol, gene title, p-value, and log fold change (logFC) values [20].

In adherence to stringent criteria, genes meeting the thresholds of a p-value < 0.05 and a logFC value ≥ 1 were identified as upregulated DEGs and p-value < 0.05 and a logFC value ≤ -1 were identified as downregulated DEGs across all datasets [21]. Subsequently, a critical step involved retrieving common DEGs shared among RA, IBD, and dementia which was facilitated by Venn analysis, executed through the Venny web tool (v2.1.0; https://bioinfogp.cnb.csic.es/tools/venny/), providing a foundational basis for downstream analyses. This systematic approach ensures a standardized and coherent methodology in the identification and comparison of DEGs across diverse conditions.

### 2.3 Construction and analysis of Gene-Gene Interaction (GGI) network

The interactions of the DEGs with medium confidence were methodically investigated using the STRING interaction database, (version 11.0; https://string-db.org/), after which the Gene-Gene Interaction (GGI) network was both visualized and analyzed through the utilization of Cytoscape software (version 3.10.1; https://cytoscape.org/). Following the construction of the network, the CytoHubba plugin within Cytoscape was employed to discern highly connected nodes [22]. Among the 11 ranking methods provided by CytoHubba, we selected three specific methods—Degree, Closeness, and Maximum Clique Centrality (MCC)—to pinpoint hub genes within our network [22]. The top 10 genes were identified for each of the methods mentioned above. To enhance robustness, the genes covered by all three ranking methods were deemed as hub genes, ensuring a comprehensive and reliable characterization of key molecular entities within the network [23].

### 2.4 Cluster network and functional enrichment analysis of the hub genes

The interrelationships among hub genes were comprehensively scrutinized through the application of the GeneMania plugin within the Cytoscape software (version 3.10.1) [24]. A co-expression network was systematically constructed to elucidate the functional implications of the identified hub proteins. Subsequently, these hub proteins underwent enrichment analysis employing the ’g: Profiler’ web server (version e110_eg57_p18_4b54a898; https://biit.cs.ut.ee/gprofiler/gost), facilitating the delineation of pertinent Biological Processes (BP), Molecular Functions (MF), Cellular Components (CC), and pathways, using the FDR adjusted p-value < 0.05 as the threshold. Additionally, an investigation was conducted into the expression patterns of hub proteins across distinct immune cells, utilizing data from the Human Protein Atlas Database (https://www.proteinatlas.org/). This multifaceted approach provides a nuanced understanding of the hub proteins’ functional roles, their involvement in specific pathways, and their expression profiles in various immune cell populations.

### 2.5 Gene regulatory network analysis

The regulatory networks, predominantly shaped by transcription factors (TFs) and microRNAs (miRNAs), represent pivotal components in cellular processes [25]. To unravel the regulatory dynamics surrounding the hub genes, the TRRUST database (https://www.grnpedia.org/trrust/) was used to predict transcription factors interacting with these central nodes. Complementing this, an assessment of interaction types was conducted, leveraging literature evidence for comprehensive insights. Concurrently, miRDB (https://mirdb.org/) was utilized to forecast interactions between hub genes and miRNAs, focusing on miRNAs with a target score exceeding 80. The resulting transcription factor-hub gene and miRNA-hub gene interaction networks were constructed and visually represented using the Cytoscape software (version 3.10.1), providing a systematic and integrative view of the regulatory landscape governing the hub genes within the broader context of gene expression control.

### 2.6 Drug- hub gene interaction analysis

In this investigation, we leveraged the Drug Gene Interaction Database (DGIdb), an online repository (https://www.dgidb.org/) dedicated to understanding the reported interactions between hub genes and established pharmaceutical agents. Also using KEGG pathway enrichment analysis, we identified common pathways involved in RA, IBD, and dementia. Following extracting pertinent information from DGIdb and identifying the drugs targeting the shared pathways and the regulatory elements, we constructed an interaction network, which was subsequently subjected to comprehensive visualization and analysis utilizing Cytoscape software version 3.10.1. The drugs targeting multiple pathways or important regulatory hubs were prioritized in the network analysis. This approach allows for a systematic delineation of potential associations between hub genes -existing drugs, and identification of drugs targeting overlapping mechanisms contributing valuable insights to the broader understanding of pharmacological implications within our study’s context and diving deeper into the properties of the molecules explored extensively.

## 3. Results and discussion

### 3.1 Data curation for the study

The study of comorbidities, the concurrent presence of multiple medical conditions in a single individual alongside a primary disease, offers essential insights for understanding, managing, and treating complex diseases. Comorbidities can significantly affect the progression, severity, and outcomes of a primary disease by introducing additional underlying processes that may either exacerbate or alter the course of the disease [26]. This study, aimed to identify shared biomarkers among RA, IBD, and dementia through a thorough analysis of gene expression datasets sourced from the NCB1-GEO database. Using the specific keywords and the inclusion criteria detailed in section 2.1, we curated and analyzed a set of ten microarray gene expression datasets of RA, ten for IBD, and nine for dementia.

### 3.2 Identification of the common DEGs between RA, IBD, and dementia

**Fig 2:**
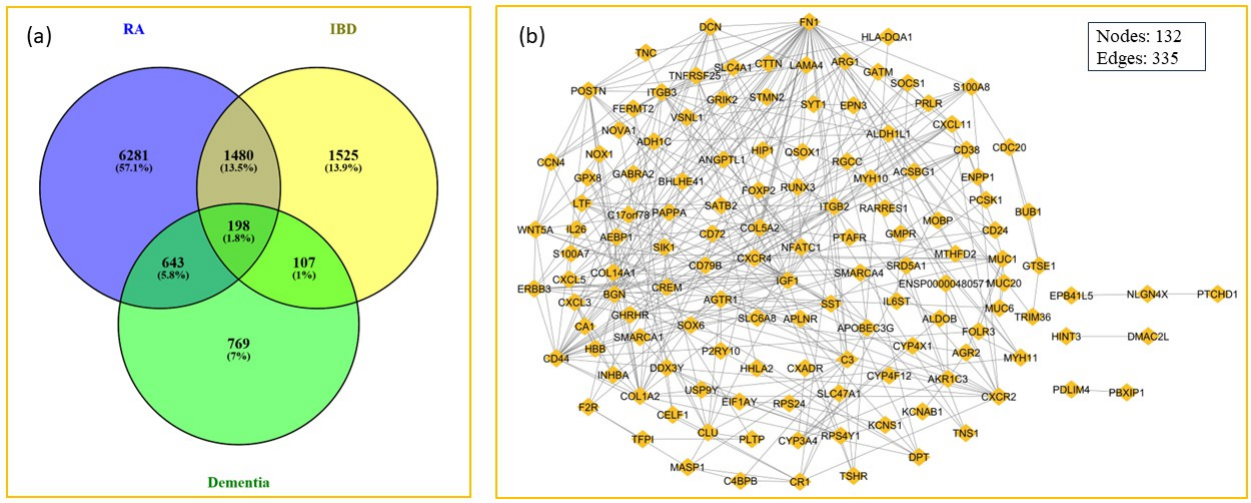
**(a)** The unique and shared DEGs across RA, IBD, and dementia. **(b)** Network of the common DEGs of RA, Dementia, and IBD. The nodes depict the genes and the edges represent the interaction between the nodes.

Comparative analyses were conducted between control and case samples to extract DEGs for further scrutiny as these genes show the altered gene expression between the healthy and diseased states. We applied ComBat to correct batch effects and utilized the limma package to identify the DEGs, a widely used and validated tool for microarray data analysis, particularly in cases of small sample sizes or variation across platforms. The DEGs were filtered based on a significant threshold set at a p-value < 0.05 and a log fold change (FC) ≥ 1 for upregulated DEGs, while downregulated DEGs were defined by a p-value < 0.05 and an FC ≤ -1. We identified 9093 DEGs in RA datasets, comprising 2676 upregulated and 6417 downregulated genes. The IBD datasets yielded 3358 DEGs (2,053 upregulated and 1,305 downregulated), while dementia datasets revealed 1,766 DEGs (934 upregulated and 832 downregulated) (S2–S4 Tables in S1 File). Following the initial analysis, we employed the Venny web tool to perform a comparative analysis across these three conditions, identifying 198 DEGs shared between RA, IBD, and dementia datasets (Fig 2a). This subset of shared DEGs was prioritized for further investigation into the molecular overlap across these diseases, seemingly pointing toward the shared underlying molecular mechanisms.

### 3.3 GGI network analysis and hub gene determination

We systematically examined the proteins encoded by the identified DEGs to construct a comprehensive GGI network. We employed the STRING database version 11.0, to generate the network which was then visualized and analyzed using Cytoscape software version 3.10.1. After removing the duplicates and non-interacting proteins, the final GGI network consisted of 132 nodes and 335 edges, as depicted in Fig 2b. The GGI network provides insight into the connected nature of proteins, which might be part of shared pathways or key regulators.

**Fig 3:**
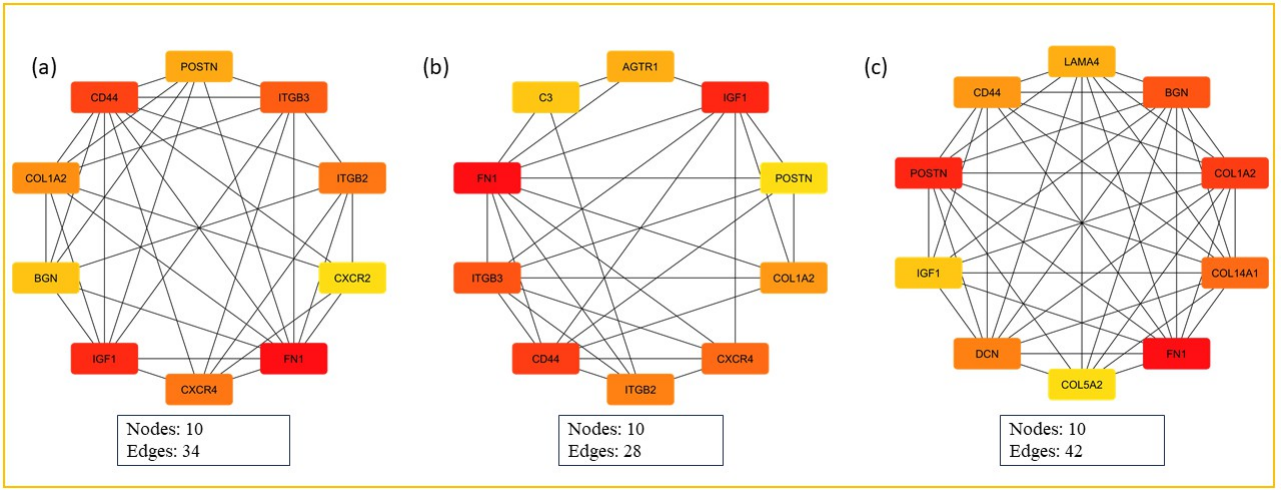
The topological analysis of the PPI network (a) Degree (b) Closeness (c) MCC.

**Fig 4:**
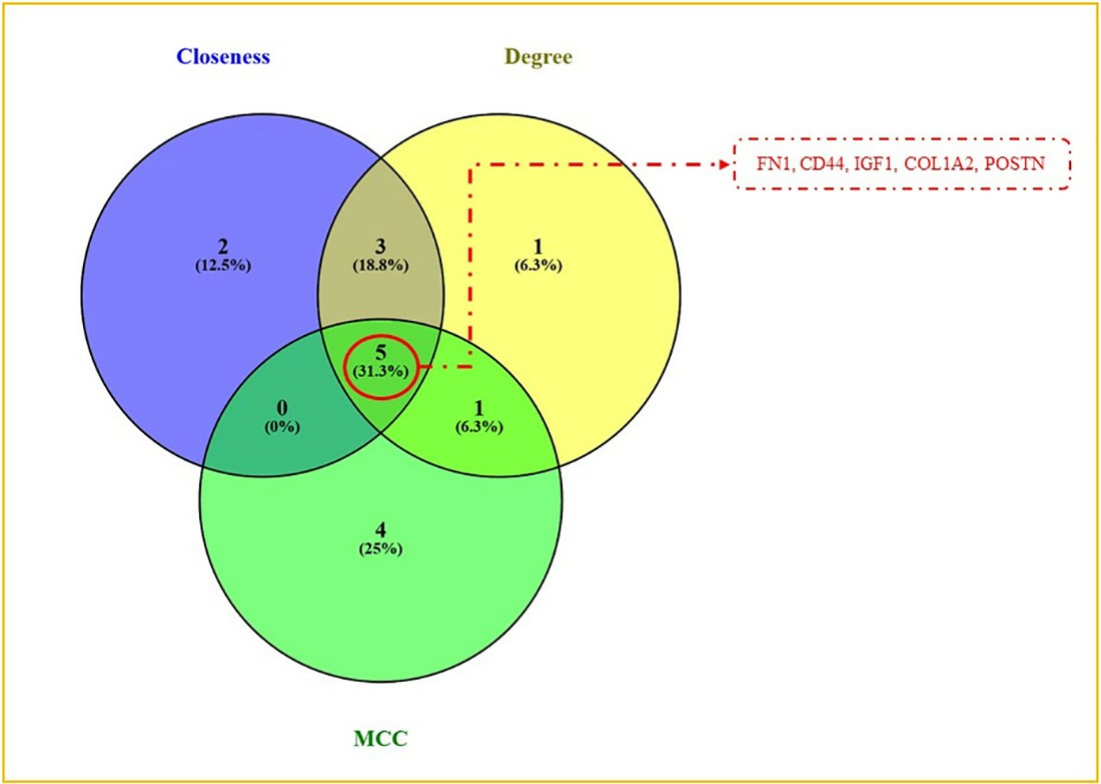
Hub gene identification. The unique and shared genes across the topological features degree, closeness, and MCC.

To ascertain the network’s topological features, we performed an in-depth analysis using the CytoHubba plugin within Cytoscape, incorporating measures of closeness, degree, and MCC (Fig 3a–3c) (Table 1). This topological analysis of the top 10 genes identified five hub genes- *FN1*, *CD44*, *IGF1*, *COL1A2*, and *POSTN* each consistently ranked among the top genes across the three centrality measures, as shown in Fig 4. A detailed description of the hub genes and their involvement across all three diseases is presented in S5 Table in S1 File, providing comprehensive insights into their potential as key biomarkers addressing the interconnected pathology of RA, IBD, and dementia.

**Table 1:** Topological feature analysis for the hub genes extracted.

Sl No	Degree	Closeness	MCC
**1**	*FN1*	*FN1*	*FN1*
**2**	*IGF1*	*IGF1*	*POSTN*
**3**	*CD44*	*CD44*	*COL1A2*
**4**	*ITGB3*	*ITGB3*	*BGN*
**5**	*CXCR4*	*CXCR4*	*COL14A1*
**6**	*ITGB2*	*ITGB2*	*DCN*
**7**	*COL1A2*	*COL1A2*	*CD44*
**8**	*POSTN*	*AGTR1*	*LAMA4*
**9**	*BGN*	*C3*	*IGF1*
**10**	*CXCR2*	*POSTN*	*COL5A2*

To extend our investigation of these hub genes’ potential roles across RA, IBD, and dementia, we examined the specific functions and pathogenic connections of the hub genes. While the role of Fibronectin 1 (FN1) in dementia remains under investigation, emerging studies in RA propose a close association between FN1 and the initiation and progression of the disease [27]. This connection is further supported by Al-Numan et al., who identified a rare genetic variant (G313V) in FN1 associated with the early onset of IBD through exome sequencing studies, highlighting its significance in inflammatory pathology [28]. CD44, recognized as a receptor for matrix metalloproteinases (MMPs), is similarly implicated in RA, IBD, and neurodegenerative disorders, demonstrating its essential role in mediating inflammatory processes in these conditions [29, 30]. Another pivotal hub gene, IGF1, has established roles in immune modulation and inflammatory response. Acting as a lymphohematopoietic cytokine, IGF1 promotes T-cell proliferation and survival through AKT signaling, engaging deeply with immune cells [31]. Independent studies by Lee et al and Erlandsson et al reveal elevated IGF1 and IGF1BP3 levels in active RA cases, suggesting a correlation between IGF1 and IL-6 expression [32, 33]. Inhibition of the IGF1 receptor (IGF1R) notably reduces IL-6 levels, mediated by transcription factors NFKB1 and RELA, suggesting IGF1’s impact on inflammatory cascades [32, 33]. Although IGF1 levels varied across the diseases examined, a consistent pattern was not observed, indicating that IGF1’s influence may be contingent on specific disease activity and context [34]. In contrast, while limited reports implicate COL1A2 in RA, IBD, and dementia, isolated findings reveal distinct expression patterns that may influence disease progression. RA studies suggest insufficient expression of type I collagen, encoded by COL1A2, which contributed to bone loss, whereas IBD-associated COL1A2 overexpression may exacerbate intestinal collagen degradation [35]. Experimental evidence has shown the involvement of COL1A2 as a component of the extracellular matrix, which is important in maintaining blood-brain barrier integrity and contributes actively to the neurodegenerative processes [36, 37]. The varied expression of COL1A2 reflects its complex involvement in RA, IBD, and dementia pathophysiology. Finally, periostin (POSTN), an extra-cellular matrix protein with roles in bone metabolism, is observed to influence WNT signaling pathways crucial for osteoblast differentiation and bone maintenance by suppressing apoptosis in precursor cells. The under expression of POSTN in RA patients suggests suppression of WNT activity, potentially impairing bone health. Conversely, POSTN overexpression in IBD has been linked to inflammatory processes via the NFKB pathway [38, 39]. Intriguingly, POSTN downregulation in Alzheimer’s disease (AD) suggests it may have neuroprotective properties, with reduced expression potentially leading to impaired axonogenesis and neuronal support [40]. Together these findings underscore the multifaceted roles of these hub genes across RA, IBD, and dementia, highlighting their potential as biomarkers and therapeutic targets.

### 3.4 Cluster and functional enrichment analysis

**Fig 5:**
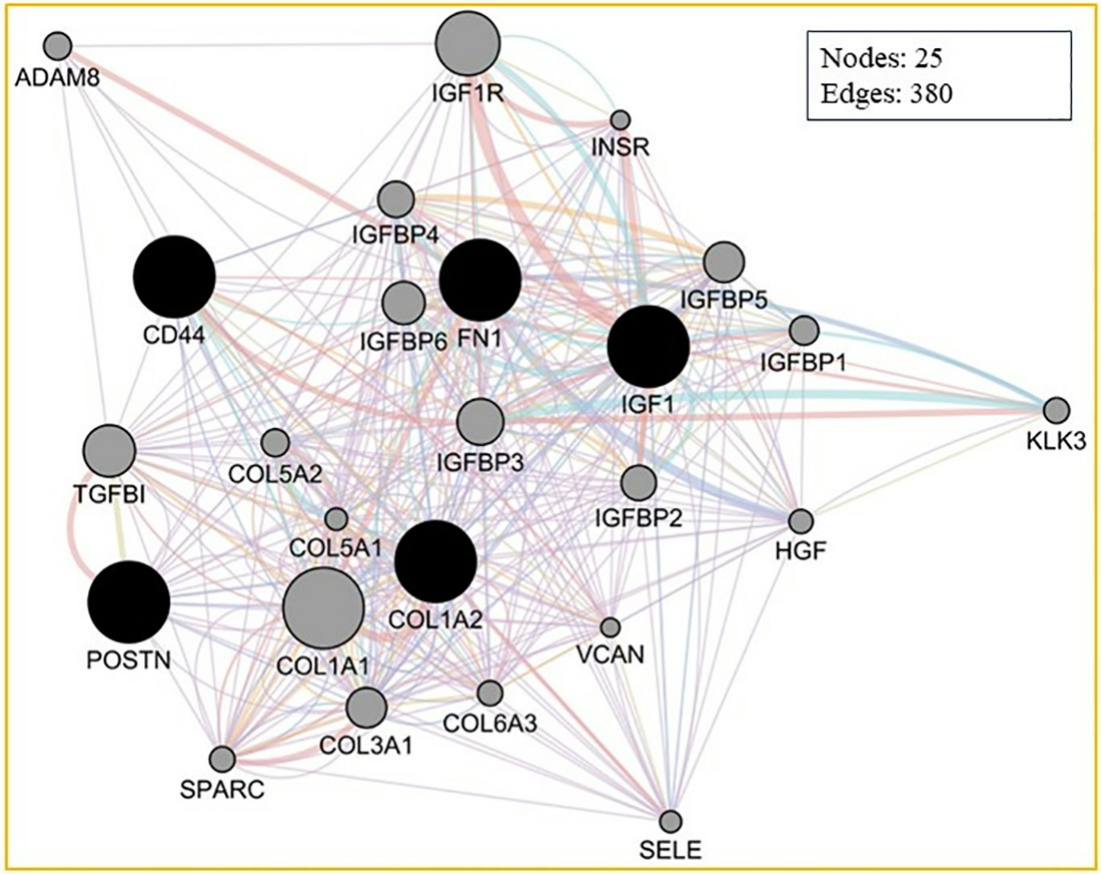
Gene cluster interaction network: The edges in lilac show co-expression, red edges show physical interactions, yellow edges depict shared protein domains and the blue edges code for pathways.

To understand the interaction dynamics among the hub genes, we performed a cluster analysis to characterize gene-gene interaction, revealing a broad spectrum of functional associations. This encompassed co-expression, predictive functional associations, direct physical interactions, shared protein domains, and co-localization [24]. Grouping the hub genes by interaction patterns enabled us to explore functionally significant relationships with the network. The resulting interaction network, visualized in GeneMania, displayed 25 nodes interconnected by 380 edges (Fig 5). The weighted analysis of the interaction network indicated that physical interaction comprised the largest proportion at 74.59%, followed by co-expression at 16.57%, co-localization at 3.32%, shared protein domain at 0.57%, and predicted functional relationship at 0.36% (S6 Table in S1 File), suggesting that physical interaction is the dominant association among hub genes, highlighting the potential of these genes to influence each other within shared biological pathways. The significant co-expression levels also imply their participation in the synchronized regulatory mechanism across the diseases.

Additionally, an extensive gene ontology (GO) and KEGG analysis was conducted for the hub genes. The significant BP, CC, and MF identified are detailed in S7 Table in S1 File and illustrated through bubble plots in (S7 Table in S1 File; S1 Fig). The pathway enrichment analysis underscored the involvement of critical pathways, notably proteoglycans in cancer, extracellular matrix (ECM) receptor interaction, focal adhesion, PI3K-AKT signaling, AGE-RAGE signaling, and amoebiasis underscoring the functional roles of the hub proteins (S7 Table in S1 File). The GO and KEGG pathway enrichment of the hub genes clarify their involvement in critical molecular functions and pathways associated with the pathology of RA, IBD, and dementia. Particularly, the identified functions of collagen and glycosaminoglycan (GAG) binding, show their potential roles in structural support and inflammatory processes, central to RA pathology and likely relevant to other inflammatory processes in IBD and dementia [41]. The analysis also highlighted biological processes related to cell adhesion regulation and the ERK1 and ERK2 signaling cascades, both known to offer cellular responses in inflammation and tissue remodeling, which are important in the advancement of these diseases. Examination of the top cellular components stressed the extracellular space and matrix, which are the key regions of interaction between inflammatory mediators and structural proteins, influencing tissue integrity. Importantly, enriched pathways such as focal adhesion, PI3K-AKT pathway, and ECM-receptor interaction pathways are, heavily implicated in all three diseases, aligning with their known involvement in cell survival, migration, and differentiation. ECM receptor interaction pathways and focal adhesion pathways are implicated in tissue integrity, cell migration, and immune responses, while the PI3K-AKT pathway has a dual role in cell survival and apoptosis [42, 43]. These are also highly connected in sustaining inflammatory mediators, devising structural changes across joints, gut, and neural tissues, suggesting shared molecular mechanisms. The shared genes within these pathways might serve as potential prognostic biomarkers and targeting them may help predict therapeutic responses and early detection of disease progression or complications. These common regulatory elements are of therapeutic relevance as drugs such as PI3K inhibitors are already in clinical trials for other conditions like cancer, which could be repurposed for RA, IBD, and dementia, given the pathway has shared involvement [44, 45].

**Fig 6:**
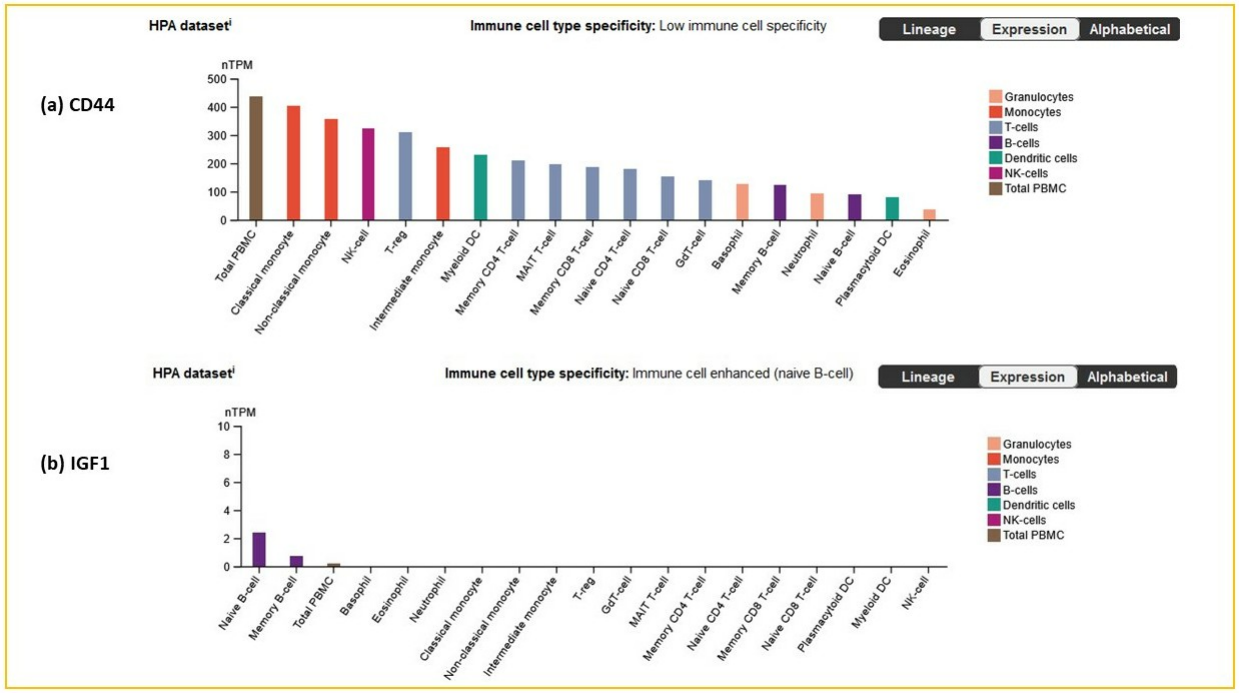
Immune cell expression. Hub gene enrichment in the immune cells, where (a) CD44, and (b) IGF1 gene enrichment in the immune cells are shown.

Further analysis of hub protein expression across immune cell types revealed notable enrichment of CD44 across a wide range of immune cell classes. This broad expression of the CD44 across immune cells aligns well with its known role in mediating cell-cell interactions, which is critical in autoimmune diseases. In contrast, IGF1 demonstrated marked expression in naïve B-cells, suggesting a more targeted role in early-stage immune cell activation or differentiation suggestive of its role in disease initiation or immune homeostasis (Fig 6). Remarkably, FN1, COL1A2, and POSTN exhibited negligible expression across all immune cells, implying that their role may be more involved in tissue-specific processes such as extracellular remodeling than direct immune modulation. The broad expression of CD44 across immune cells aligns with its known role in mediating cell-cell interactions.

### 3.5 Determination of regulatory networks of hub genes

**Fig 7:**
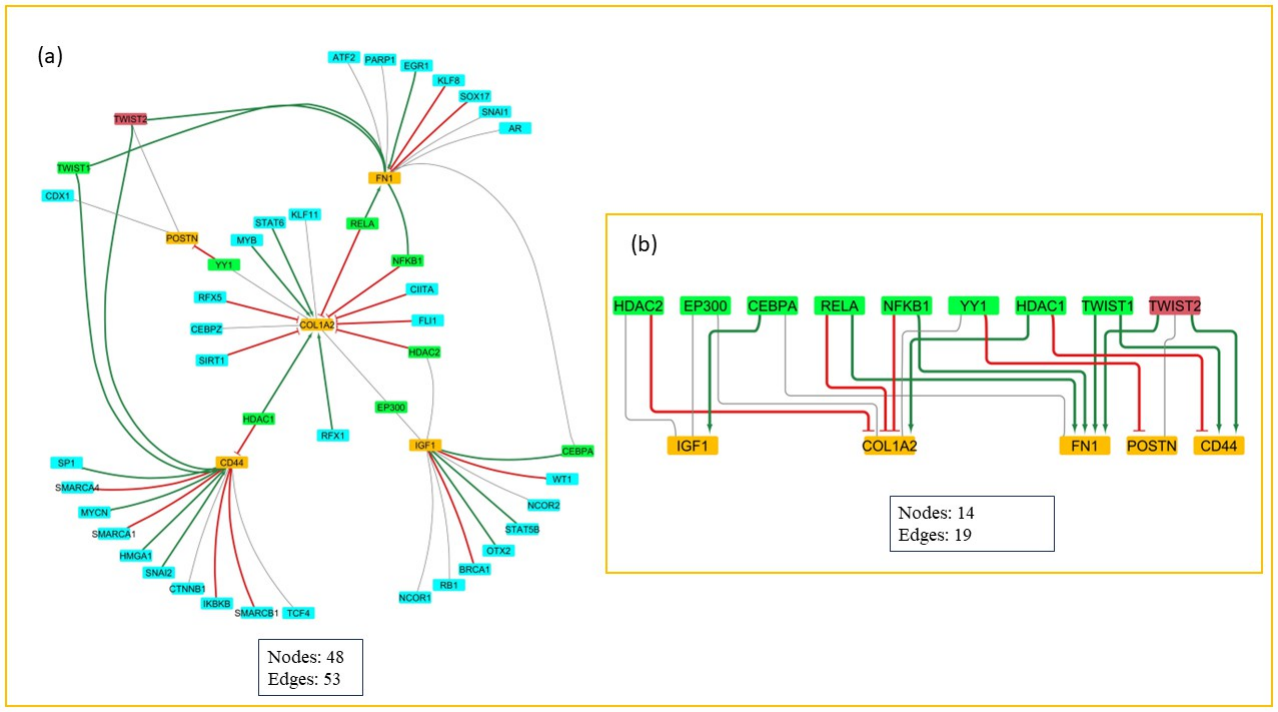
**(a)** TF-hub gene network. The interaction network of the identified hub genes and the transcriptional factors. **(b)** Hub TF retrieval. Interaction network of the key transcription factors. The yellow nodes are the hub genes, green and red nodes are the key transcription factors.

In this study, we identified regulatory TFs and miRNAs linked to hub proteins, establishing a comprehensive regulatory network that provides insights into post-transcriptional and translational control mechanisms governing the pivotal proteins. The resulting TF-hub protein interaction network, depicted in Fig 6a, displays hub proteins as yellow rectangles, while transcription factors regulating more than two hub genes are marked in red, those regulating at least two hubs appear in green and the others are shown in aqua. The regulatory interactions are represented by edges, where red denotes inhibitory effects, green indicates activation, and grey signifies interactions with limited documentation (Fig 7a) (S8 Table in S1 File). Key regulatory transcription factors identified through degree score analysis (≥2) included TWIST2, CEBPA, EP300, HDAC1, HDAC2, NFKB1, RELA, TWIST1, and YY1 all of which demonstrate pivotal regulatory influence over multiple hub proteins (S9 Table in S1 File) (Fig 7b). In examining key hub genes, we delineated the associated upstream TFs and miRNAs, identifying a network of prominent regulators. Our analysis underscores the substantial regulatory roles of RELA and NFKB1, the central elements of the NF-κB signaling pathway known to fuel the inflammatory responses and implicated in RA, IB, and dementia pathogenesis [46, 47]. This regulatory network integrates multiple inflammatory pathways, where the NF-κB pathway, closely interacting with TGFβ, WNT, and ERK signaling cascades, facilitates chronic inflammation and immune dysregulation, which are the hallmarks of these diseases [48]. Furthermore, HDAC1/2 and EP300, key factors in the TGFβ pathway, enhance these regulatory effects by modulating pathways that affect cellular adhesion and matrix integrity, with potential downstream impacts on tissue integrity and immune cell migration [48]. The co-involvement of HDAC1/2, NFKB1, and RELA in neutrophil extracellular trap formation, also emphasizes a shared mechanism that promotes increased expression of inflammatory mediators, disrupts epithelial barrier function, and accelerates extracellular matrix degradation, all of which marks central to the progression of all these conditions [49]. The enrichment of multiple core TFs (CEBPA, RELA, NFKB1, HDAC1/2, EP300) in cancer-related pathways suggests an intriguing overlap, as both RA and IBD are increasingly recognized to mimic tumor microenvironment [50]. This insight reinforces the potential role of these TFs in disease pathophysiology, where chronic inflammation and altered cellular environments may create pro-tumorigenic conditions.

**Fig 8:**
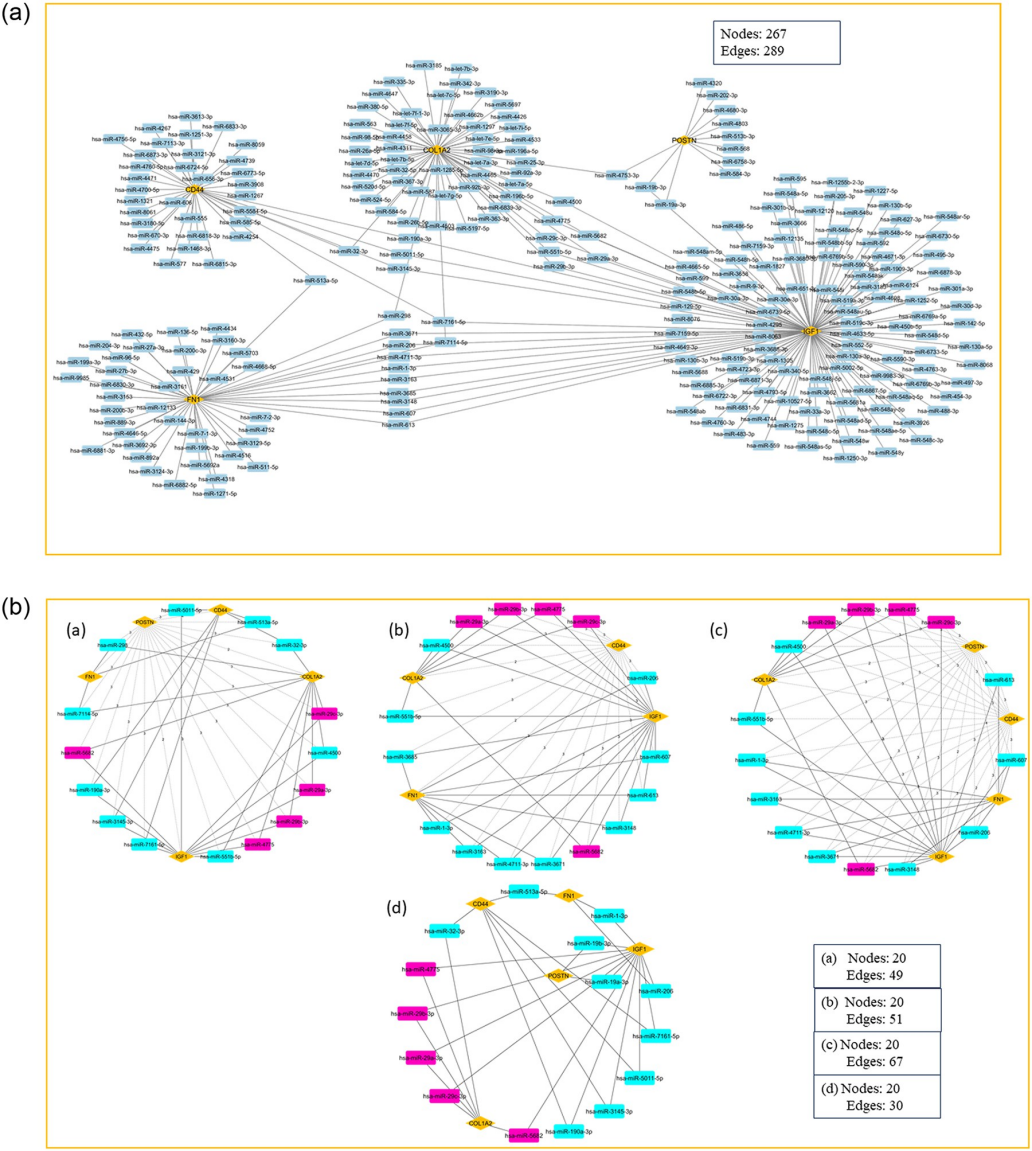
**a:** The miRNA-hub gene interaction network, where the hub genes are denoted in yellow and the miRNAs are shown in blue nodes. **b:** Hub miRNA retrieval. The hub miRNAs predicted in our study through topological analysis using the (a) Betweenness, (b) Closeness, (c) Stress, and (d) Degree of the CytoHubba plugin of Cytoscape. The yellow color diamond nodes are the hub genes, the blue square nodes are the predicted miRNA, and the pink nodes are the predicted miRNAs that are common among all the four topology measures.

Our analysis uncovered 289 distinct miRNAs that collectively govern the regulatory landscape of the hub proteins (S10 Table in S1 File). Through visualization and network analysis using Cytoscape V3.10.1, we constructed a miRNA-hub protein interaction network, identifying IGF1 as the hub gene with the highest miRNA interactions, followed closely by COL1A2, both exhibiting extensive regulatory links (Fig 8a). Although no single miRNA was observed to regulate all five hub genes, IGF1 and COL1A3 displayed the most shared miRNA interactions among the hub genes. To determine the key regulatory miRNAs, we applied four centrality ranking algorithms- betweenness, closeness, degree, and stress, to identify the top 20 miRNAs, with those recurring across all designated as hub miRNAs (Fig 8b; S11 Table in S1 File). This approach revealed five core miRNAs: hsa-miR-4775, hsa-miR-29a-3p, hsa-miR-29b-3p, hsa-miR-29c-3p, and hsa-miR-5682. These miRNAs exhibit significant interactions with both IGF1 and COL1A2, positioning them as central regulatory elements in our network. Although the role of hsa-miR-5682 is not elucidated in human diseases, hsa-miR-4775 has been well characterized in promoting colon cancer invasion and metastasis through the Smad7/TGFβ axis, suggesting its possible involvement in similar signaling pathways relevant to inflammatory diseases [51]. Multiple experimental studies on patient cohorts highlight the miR-29 family (miR-29a, miR-29b, and miR-29c) as critical regulators in the network of inflammatory processes shared by RA, IBD, and dementia. The miR-29 family emerged as an important regulatory axis, with its known anti-inflammatory role documented in RA fibroblast-like synoviocytes (RA-FLS), where miR-29a is downregulated while miR-29b, possibly under the influence of TNF-α, is upregulated, in turn suggesting that miR-29 family members are responsive to inflammatory signals in RA [52]. Infliximab-treated RA patients showed a repressed expression of miR-29 contributing to increased apoptotic resistance of peripheral blood mononuclear cells, through inhibiting HBP1 signaling involved in the regulation of cell cycle and apoptosis, leading to the sustained inflammation in RA [53]. Furthermore, the predictive values of miR-29, miR-26b, and miR-451 levels for olokizumab therapy response emphasize the therapeutic relevance of these miRNAs in personalized treatment strategies, although the findings currently are population-specific [52, 54]. Experimental studies have shown that elevated mir-29 levels in active ulcerative colitis and Crohn’s disease suggest that these miRNAs may play a central role in promoting inflammation by modulating the IL-23 expression, an inflammatory cytokine associated with all three diseases examined [55]. The IL-23 cytokine is especially indicated in activating Th17 cells, which are involved in the inflammatory cascades. Moreover, the association of IL23R variants with increased IBD susceptibility adds further weight to the role of the miR-29-IL23 axis in linking these conditions [56]. miR-29 has various roles in dementia pathology which include neurodevelopment, amyloid pathology, epigenetics, and neurodegeneration. Through epigenetic control of non-CG DNA methylation, miR-29 is significant to brain maturation and neuronal health, and any dysregulation of miR-29 in early life is seen as a predisposition to dementia [57]. Other studies have also reported that miR-29 disrupts the regulation of BACE1, leading to elevated amyloid plaque formation and accelerated cognitive decline [58]. miR29 acts as a bridge between early-life neurodevelopment and late-life neurodegeneration. Though the loss of the miRNA is reported detrimental, the explicit modulation could have therapeutic potential as per studies in animal models [59].

**Fig 9:**
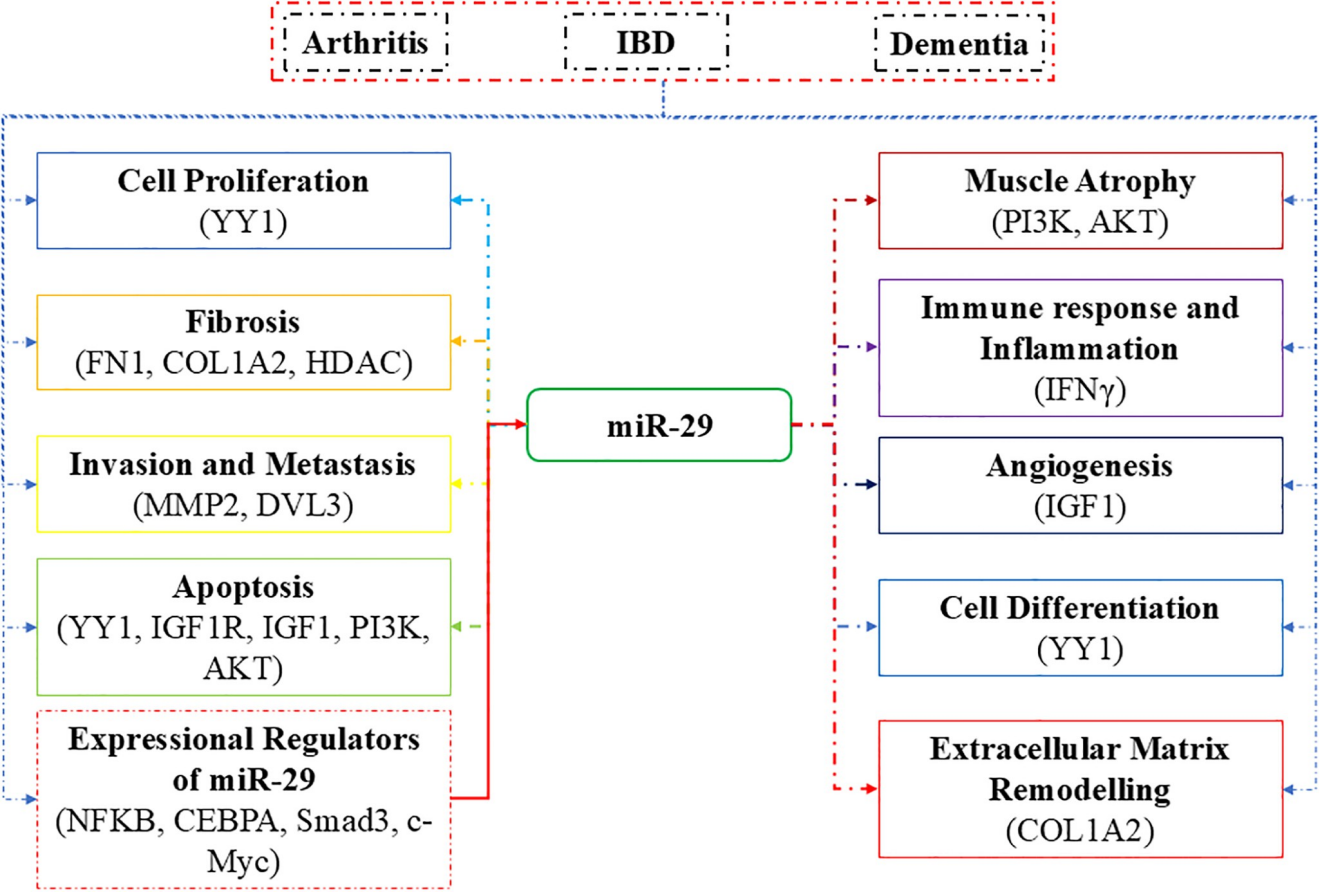
Expressional regulators and the target genes of miR-29 involved in the subprocesses associated with RA, IBD, and dementia.

These findings support the notion that the miR-29/Il-23 axis, through its regulation of hub genes, transcription factors, and inflammatory pathways, may represent a shared molecular mechanism underlying RA, IBD, and dementia. The interconnectedness of these factors points to potential therapeutic targets that could be used for treating comorbid inflammatory conditions (Fig 9).

### 3.6 Hub-gene drug interaction network analysis

The drug-target interaction network, curated from DGIdb-sourced data, reveals a structured landscape of 38 nodes and 35 edges, as illustrated in S2 Fig (S12 Table in S1 File). Our predictive analyses identified 35 distinct drugs anticipated to interact with the hub proteins, offering potential avenues to influence their regulatory dynamics. Specifically, the CD44 hub protein emerges as a focal point, with 13 drugs predicted to modulate its activity, underscoring its pharmacological relevance. Similarly, the FN1 hub protein is identified as another critical target with 11 drugs, emphasizing the diversity of pharmacotherapeutic interventions directed toward pivotal proteins within our network. This systematic exploration of drug-hub protein interactions contributes valuable insights into potential pharmacological avenues for modulating key proteins implicated in the molecular pathways under investigation. This analysis of the drug-gene interaction network revealed several drugs with notable neuroprotective and anti-neoplastic properties, targeting pathways integral to inflammation, ulceration, neuroinflammation, neoplastic activity, and osteoporosis. This profiling opens promising avenues for drug repurposing, providing potential therapeutic interventions that could regulate key biological elements of RA, IBD, and dementia [60, 61]. Based on these findings, we identified 11 pathways common to RA, IBD, and dementia, through KEGG pathway analysis such as the NF-κB signaling pathway, PI3K-AKT pathway, JAK-STAT pathway, TGF-B signaling pathway, MAPK pathway, WNT pathway, PPAR signaling pathway, apoptosis pathways, focal adhesion pathway, oxidative stress pathway, and Th17 signaling pathway. Using these pathways, we fetched drugs that could affect these overlapping mechanisms. The identified drugs (S15 Fig in S1 File) targeting multiple pathways are highlighted which act as promising candidates for multi-disease therapeutic strategies. This analysis shows coherence with the previous results of our study, highlighting the potential for drug repurposing. These findings suggest a therapeutic approach that could address the comorbid and systemic nature of these diseases, broadening the therapeutic goals.

### 3.7 Expression validation of hub genes

**Fig 10:**
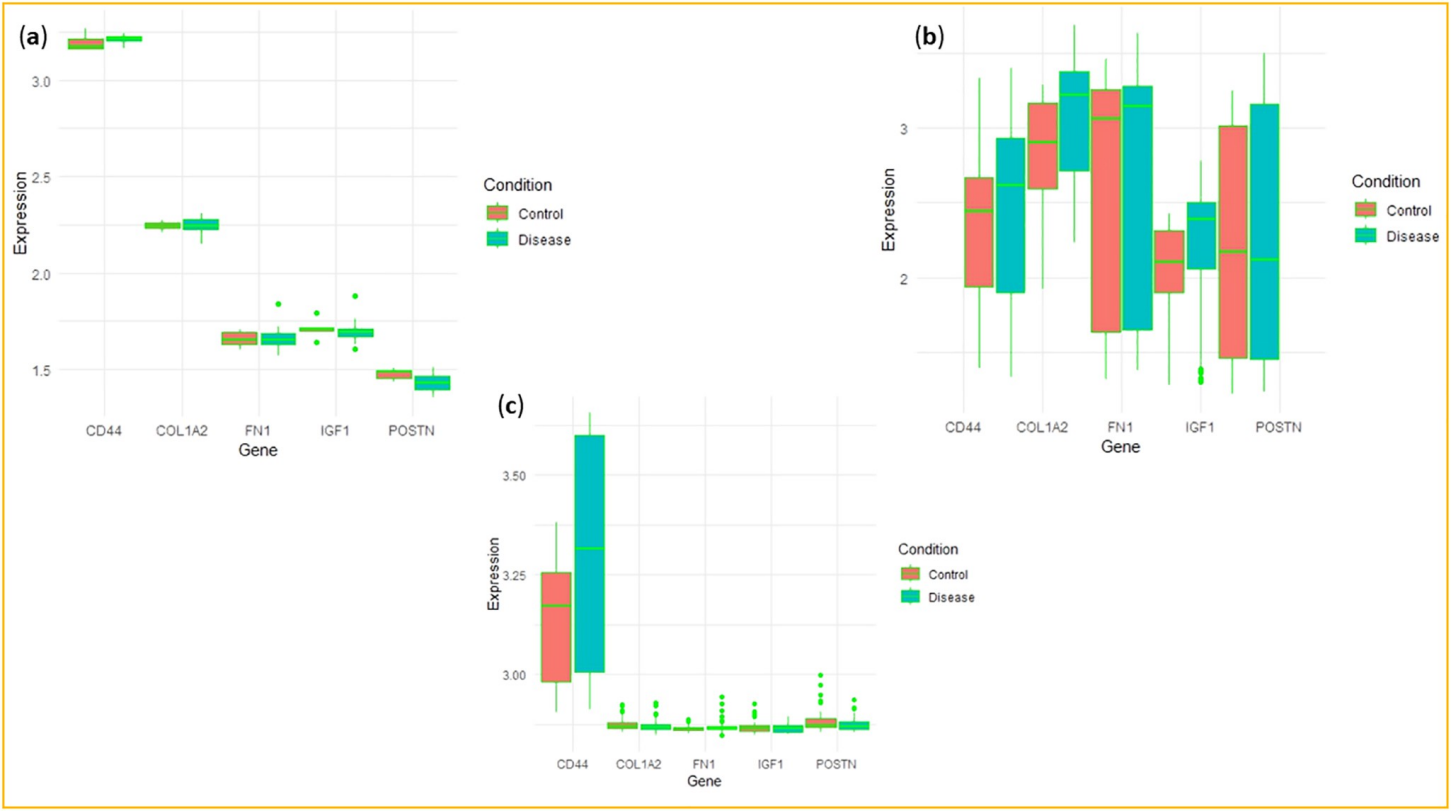
Hub gene validation. Expression level of hub genes in the validation datasets **(a)** GSE68689, **(b)** GSE9452, and **(c)** GSE140829.

Hub gene expression was validated using datasets GSE68689, GSE9452, and GSE140829, confirming differential expression patterns observed in our study. Our study found that *CD44* was upregulated and *POSTN*, *IGF1*, and *COL1A2* were downregulated in the RA sample (Fig 10a). In IBD samples, *CD44*, *FN1*, *COL1A2*, and *IGF1* were consistently upregulated, with *POSTN* showing reduced expression levels. Similarly, in dementia samples, both *CD44* and *FN1* showed increased expression, whereas *POSTN* remained downregulated (Fig 10b and 10c). The upregulation of CD44 in all three conditions again aligns with its known involvement in promoting inflammation and cell adhesion. Comparably, the increased expression of FN1 in both IBD and dementia may aim toward its role in ECM remodeling, a distinctive feature of the inflammatory and fibrotic response often associated with these diseases. Contrarily, the downregulation of POSTN in all three diseases might suggest a reverse relationship with inflammation, possibly demonstrating its role in tissue repair mechanisms that might be disrupted in these conditions.

Acknowledging the limitations inherent in our study, which predominantly employs a systems biology approach is imperative. One notable constraint arises from the diverse tissue origins of the diseases under consideration, leading to inherent tissue-dependent variations in gene expression. To mitigate this limitation, we conscientiously incorporated datasets encompassing diverse tissues, including blood, synovium, colon, and brain biopsy samples. Despite this effort, it is crucial to recognize that tissue origin variations may influence our findings’ generalizability. Additionally, the considerable number of DEGs in our study raises the potential for false positives. To address this concern, we implemented normalization, and topological analyses to identify and prioritize relevant DEGs, enhancing the reliability of our results. While microarray data provided valuable insights into this gene expression study, it is important to acknowledge that RNA-sequencing technology offers superior depth and coverage, which offers additional resolution. A substantial proportion of the downstream regulators of the hub genes are intricately involved in the inflammatory process, making it relevant to investigate the nuanced relationship between these hub genes and the dysregulated immune cell infiltration observed in the studied diseases. While our study primarily concentrates on the computational aspect, we acknowledge the importance of direct experimental validation. As a future step, we propose qPCR-based validation of the hub genes and miR-29 cluster using patient-derived samples to confirm our findings.

## 4. Conclusion

Our study aimed to unravel the intricate molecular connections and crosstalks between RA IBD and dementia, focusing on shared genetic and regulatory mechanisms that may underlie these comorbidities. Through an integrative systems biology approach, incorporating differential gene expression analysis, identification of hub genes, and other comprehensive network analyses, we identified critical TFs (RELA, NFKB1, TWIST1/2, HDAC1/2, CEBPA, EP300, and YY1) and key miRNAs (particularly hsa-miR-29a, b, c) pivotal to the regulatory landscape of these diseases. Notably, the miR-29/IL-23 axis emerged as a potential unified biomarker and therapeutic target of RA, IBD, and dementia. Additionally, the PI3K-AKT and WNT pathways, both regulated by miR-29, were majorly involved in the pathophysiology of all three diseases. By elucidating the molecular intricacies and commonalities among these diseases, our research lays the crucial foundation for precision medicine, leading to targeted, cross-disease therapeutic strategies that use these molecular overlaps for improved patient outcomes.

## Supporting information

S1 Fig The Gene ontology categories in biological process, molecular functions, and cellular components. (TIF)

S2 Fig The Drug- hub gene interaction. (TIF)

S1 File Supplementary tables. (XLSX)
