# Supplementary figures and images for: Gene expression analysis reveals mir-29 as a linker regulatory molecule among rheumatoid arthritis, inflammatory bowel disease, and dementia: Insights from systems biology approach
Source: PLoS One. 2025 Jan 15;20(1):e0316584. doi: 10.1371/journal.pone.0316584 (PMC11734936; doi:10.1371/journal.pone.0316584)

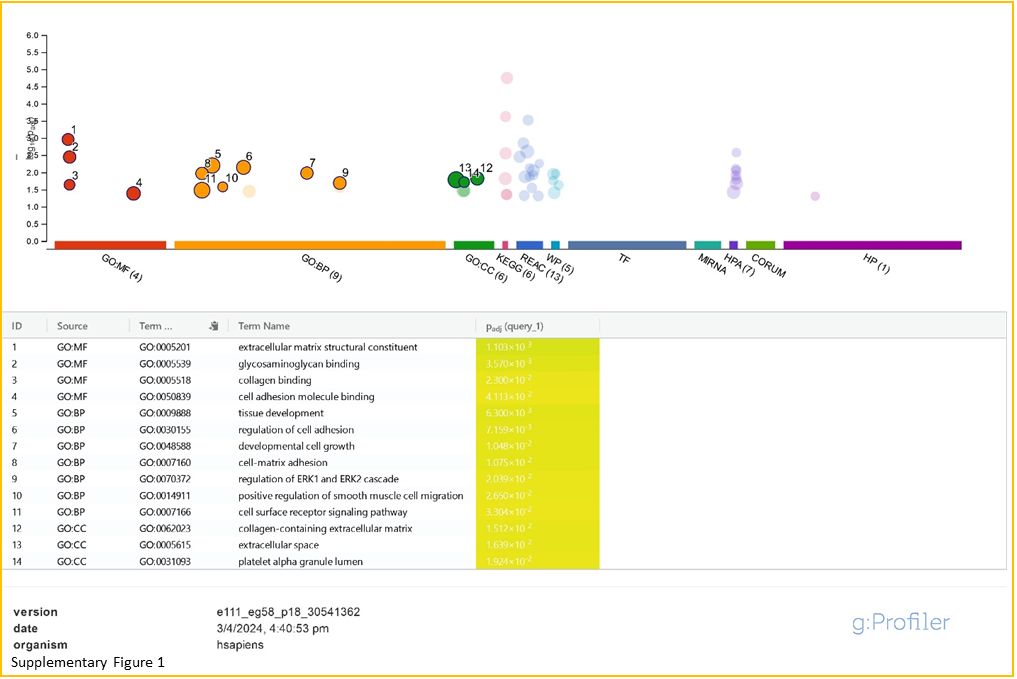

Supplement: S1 Fig — (TIF) [file pone.0316584.s001.tif]

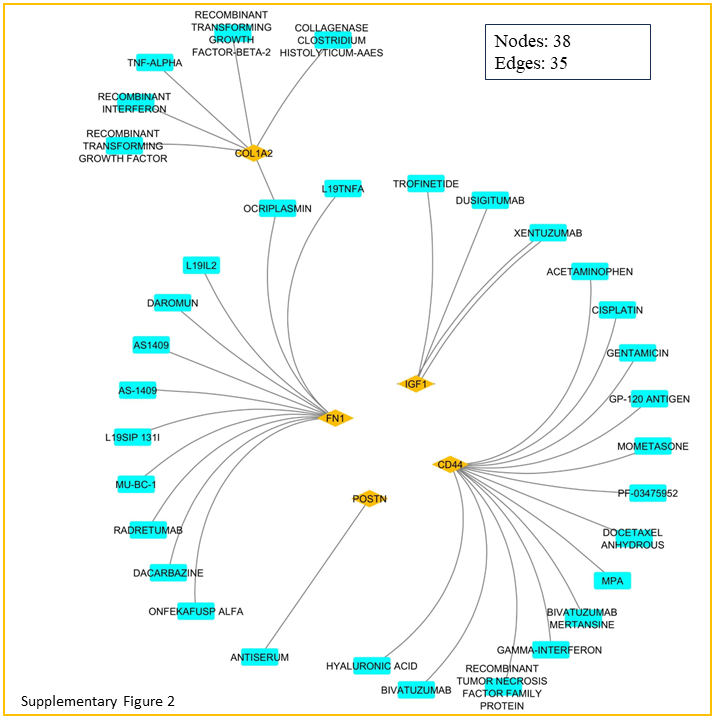

Supplement: S2 Fig — (TIF) [file pone.0316584.s002.tif]
